# Discriminating Susceptibility of Xanthine Oxidoreductase Family to Metals

**DOI:** 10.1128/spectrum.04814-22

**Published:** 2023-07-17

**Authors:** Anne-Soisig Steunou, Marion Babot, Anne Durand, Marie-Line Bourbon, Sylviane Liotenberg, Guylaine Miotello, Jean Armengaud, Soufian Ouchane

**Affiliations:** a Université Paris-Saclay, CEA, CNRS, Institute for Integrative Biology of the Cell (I2BC), Gif-sur-Yvette, France; b Université Paris-Saclay, CEA, INRAE, Département Médicaments et Technologies pour la Santé (DMTS), SPI, Bagnols-sur-Cèze, France; University of Minnesota Twin Cities

**Keywords:** copper, cadmium, metal toxicity, CO dehydrogenase, CO_2_ reductase, xanthine dehydrogenase/aldehyde oxidoreductase, CO dehydrogenase/CO_2_ reductase, photosynthetic bacteria

## Abstract

The xanthine oxidoreductase (XOR) family are metal-containing enzymes that use the molybdenum cofactor (Moco), 2Fe-2S clusters, and flavin adenine dinucleotide (FAD) for their catalytic activity. This large molybdoenzyme family includes xanthine, aldehyde, and CO dehydrogenases. XORs are widely distributed from bacteria to humans due to their key roles in the catabolism of purines, aldehydes, drugs, and xenobiotics, as well as interconversions between CO and CO_2_. Assessing the effect of excess metals on the Rubrivivax gelatinosus bacterium, we found that exposure to copper (Cu) or cadmium (Cd) caused a dramatic decrease in the activity of a high-molecular-weight soluble complex exhibiting nitroblue tetrazolium reductase activity. Mass spectrometry and genetic analyses showed that the complex corresponds to a putative CO dehydrogenase (pCOD). Using mutants that accumulate either Cu^+^ or Cd^2+^ in the cytoplasm, we show that Cu^+^ or Cd^2+^ is a potent inhibitor of XORs (pCOD and the xanthine dehydrogenase [XDH]) *in vivo*. This is the first *in vivo* demonstration that Cu^+^ affects Moco-containing enzymes. The specific inhibitory effect of these compounds on the XOR activity is further supported *in vitro* by direct addition of competing metals to protein extracts. Moreover, emphasis is given on the inhibitory effect of Cu on bovine XOR, showing that the XOR family could be a common target of Cu. Given the conservation of XOR structure and function across the tree of life, we anticipate that our findings could be transferable to other XORs and organisms.

**IMPORTANCE** The high toxicity of Cu, Cd, Pb, As, and other metals arises from their ability to cross membranes and target metalloenzymes in the cytoplasm. Identifying these targets provides insights into the toxicity mechanisms. The vulnerability of metalloenzymes arises from the accessibility of their cofactors to ions. Accordingly, many enzymes whose cofactors are solvent exposed are likely to be targets of competing metals. Here, we describe for the first time, with *in vivo* and *in vitro* experiments, a direct effect of excess Cu on the xanthine oxidoreductase family (XOR/XDH/pCOD). We show that toxic metal affects these Moco enzymes, and we suggest that access to the Moco center by Cu ions could explain the Cu inhibition of XORs in living organisms. Human XOR activity is associated with hyperuricemia, xanthinuria, gout arthritis, and other diseases. Our findings *in vivo* highlight XOR as a Cu target and thus support the potential use of Cu in metal-based therapeutics against these diseases.

## INTRODUCTION

Metalloproteins have a central role in biology, since the activity of many metalloenzymes is essential to life. Nevertheless, an excess of metal ions can cause toxicity. Intracellular competition between metal ions can give rise to mismetalation or interactions between ions in the active site that can ultimately lead to the inactivation of the enzyme (1, 2). The molybdenum cofactor (Moco)-, iron-sulfur cluster 2Fe-2S-, and flavin adenine dinucleotide (FAD)-containing xanthine oxidoreductases (XORs) are widely distributed from bacteria to mammals and play a key role in many catabolisms (3–6). Within the XOR family, xanthine dehydrogenase (XDH) catalyzes the oxidative reaction of hypoxanthine to xanthine and the reaction from xanthine to urate, while aldehyde oxidase (AO), an enzyme of broad substrate specificity, catalyzes the oxidation of aliphatic or aromatic aldehydes and various aromatic heterocycles in bacteria and eukaryotes (7). The Moco-containing carbon monoxide dehydrogenases (CODs) are bacterial enzymes that oxidize CO to CO_2_ which can be ultimately converted into biomass (8). The Escherichia coli xanthine oxidoreductase (XdhABC) is a heterotrimer with a large (81-kDa) Moco-containing subunit, a medium (31-kDa) FAD-containing subunit, and a small (17-kDa) 2×[2Fe-2S]-containing subunit (9). The amino acid sequences of the three subunits of E. coli XORs display significant similarities to enzymes of the XOR family from bacteria to mammals. Structural comparisons between mammalian XORs and the AOs of Rhodobacter capsulatus (10) and E. coli (11) revealed highly similar folds and conserved cofactor positions despite differences in subunit composition (12). The presence of Mo^2+^ and Fe-S clusters makes the XOR family susceptible to inactivation by other metal ions if the Moco or Fe-S clusters are solvent exposed and accessible. It has been reported that mouse liver tissue XDH can be inhibited by compounds containing copper (Cu^+^) or zinc (Zn^2+^) (13). Additional *in vitro* data showed that Cu^+^ or arsenic (As) can interact with XDH and inhibit bovine XDH activity (13–15). The impact of excess metal ions on XOR family function has not yet been addressed *in vivo*, despite the importance of this enzyme in the metabolism of purines and aldehydes.

In both eukaryotes and bacteria, the rigorous control of metal ion uptake and the effectiveness of efflux systems allow cells to tolerate metal excess in their immediate environment. By expelling the surplus of ions, bacteria prevent metal-derived damage (2, 16). Thus, to study the effect of metal ions on enzymes *in vivo* would normally require the use of very high metal concentrations. To overcome this constraint, mutants defective in metal homeostasis machineries, specifically in the efflux systems, can be used as models. These mutants accumulate ions within the cytoplasm and therefore help to shed light on the events that follow metal accumulation within the cell (1). The P_1_B-type ATPase family of bacterial heavy metal transporters are ubiquitous and efficient efflux pumps that extrude excessive toxic metal ions such as Cu^+^, Zn^2+^, and Cd^2+^ from the cytoplasm to the periplasm, where the metal will be detoxified by other proteins (17–20). Cu^+^-ATPase or Zn^2+^-ATPase null mutants have been used to identify the cytoplasmic targets of these metals and their mechanisms of toxicity in several bacterial species (21–24). Enzymes with exposed 4Fe-4S clusters are among the primary targets of excess metal concentrations. It was shown that Cu^+^ directly damages exposed 4Fe-4S clusters or displaces other metals from the active site (1, 21, 22). Copper can also bind nonspecifically to enzymes in the molybdenum cofactor biosynthesis pathway (25), where it can be inserted instead of molybdenum at the dithiolene moiety of molybdopterin (MPT) and therefore inhibits the activity of molybdoenzymes such as the sulfite oxidase in E. coli (9).

Here, we provide genetic and biochemical data, showing inhibition of XORs in bacteria and eukaryotes by Cu^+^ or Cd^2+^ excess. We have used mutants in which the efflux systems of Cu^+^ or Cd^2+^ were dysregulated (21, 26), leading to accumulation of Cu^+^ or Cd^2+^ in the cytoplasm, thus making it possible to measure the effect of these cations on the XOR activity. *In vitro* experiments were also performed on pure bovine milk XOR to sustain our *in vivo* findings. The human XOR activity is associated with diseases including hyperuricemia, xanthinuria, gout arthritis, and others (27, 28). Drugs used for the treatment of these diseases include inhibitors of the XOR activity. Our findings highlight the XOR family as a target of Cu and support the potential use of Cu-based therapeutics in XOR-related diseases.

## RESULTS

### Serendipitous identification of an NBT-reducing complex in Rubrivivax gelatinosus (*R. gelatinosus*).

**FIG 1:**
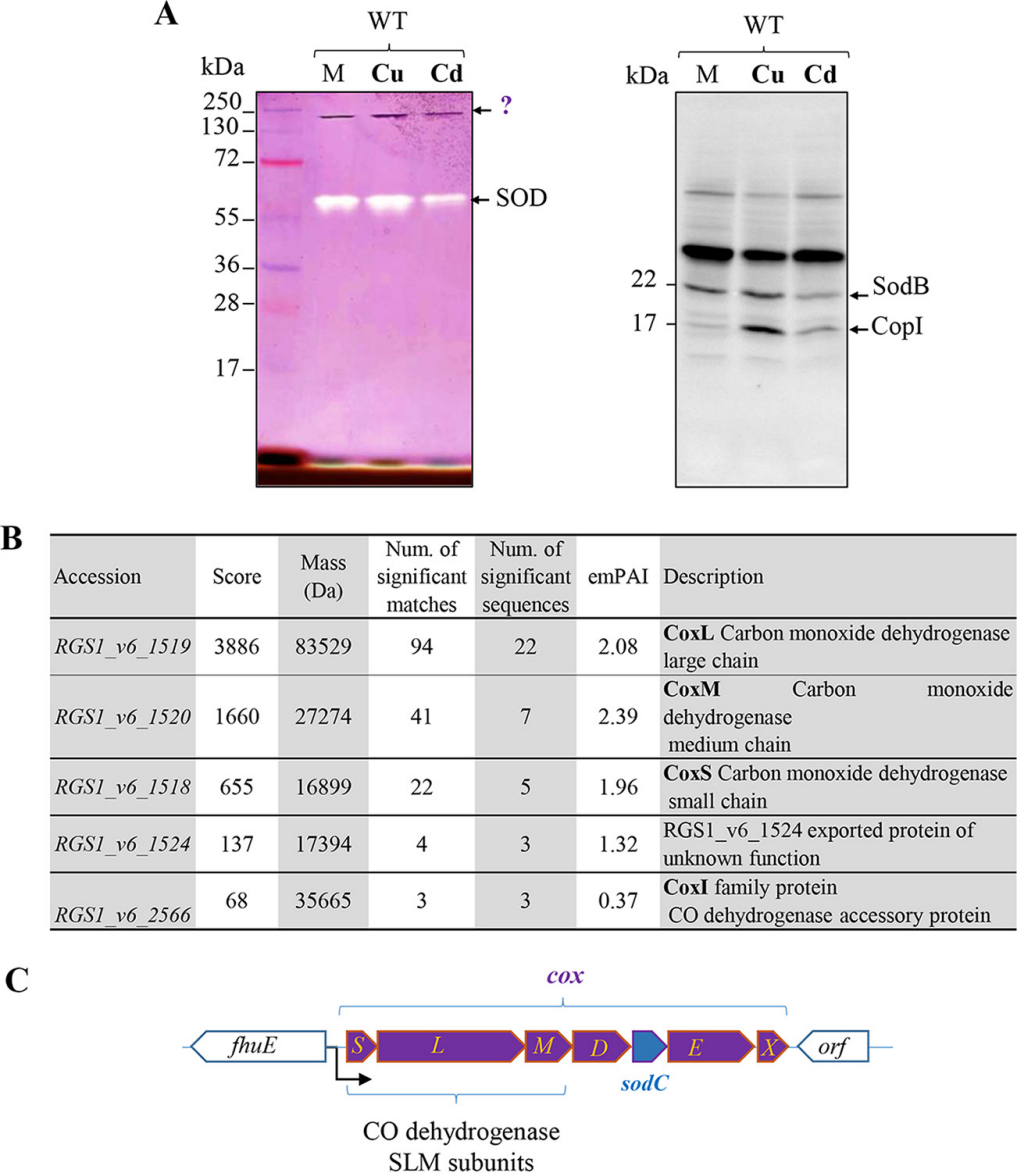
Detection of a high-molecular-weight band able to precipitate NBT and mass spectrometry identification. (A) Soluble cellular fractions of the WT *R. gelatinosus* strain grown in malate medium (M), malate medium supplemented with 100 μM CuSO_4_ (Cu), or malate medium supplemented with 100 μM CdCl_2_ (Cd) were analyzed either by native PAGE (left) or by Western blotting (right) under denaturing conditions with PAGE. The same amount (40 μg of proteins) was separated on 12% SDS-PAGE gels. After protein separation, a SOD in-gel activity assay was performed as described in Materials and Methods. SOD activity (SodB) is detected by an absence of staining, and the high-molecular-weight band is detected by an NBT-formazan deep purple precipitate. For the Western blot assay, proteins were transferred onto a polyvinylidene difluoride membrane and detection was performed using the His probe (right). (B) Mass spectrometry identification of the high-molecular-weight complex identified in panel A. Accession numbers and protein descriptions are labeled according to the fully sequenced genome of *R. gelatinosus* S1. (C) Graphic description of the *coxSLM* operon encoding the three subunits of the putative CO dehydrogenase. *fhuE* is a gene encoding an outer membrane receptor for iron uptake, *coxD* encodes an accessory protein belonging to the CO dehydrogenase complex, *coxE* encodes a protein with a CoxE-like motif, and *coxX* encodes a protein of unknown function. *sodC* encodes a putative Cu,Zn superoxide dismutase.

To examine the effect of Cu^2+^ and Cd^2+^ on the activity of the bacterial superoxide dismutase (SOD), we obtained the soluble protein fractions from wild-type (WT) *R. gelatinosus* cells grown in medium supplemented or not with excess CuSO_4_ or CdCl_2_. These protein fractions were resolved by non-denaturing PAGE and investigated using an in-gel SOD activity assay. The principle of this assay is based on the ability of superoxide to interact with nitroblue tetrazolium (NBT), reducing the yellow tetrazolium to a blue-purple precipitate within the non-denaturing PAGE gel (29). In this assay, riboflavin is used as a source of free radicals (superoxide) in the presence of light and oxygen. When a SOD is present on the PAGE gel, it appears as an achromatic band, because the SOD converts the superoxide radical into hydrogen peroxide and molecular oxygen. The remaining areas of the gel become blue-purple due to the precipitation of NBT-formazan. As shown in Fig. 1A (left), the assay showed only one achromatic band corresponding to the Fe-SOD (SodB) of *R. gelatinosus* (30) under all three tested conditions. However, an additional band of high molecular weight (between 130 and 250 kDa) that displayed a strong dark-purple staining, in sharp contrast with the SOD, was also detected. The purple band was present in all soluble fractions including those originating from cells exposed to excess metal (Fig. 1A). This dark purple color demonstrates that this unknown protein or complex can actively reduce NBT to NBT-formazan and suggests that it can generate superoxide radicals. As a proof of the Cu^+^ and Cd^2+^ stress on *R. gelatinosus* cells, a Western blot (Fig. 1A, right) showed the induction of CopI (a periplasmic protein involved in Cu and Cd homeostasis) by these ions as previously reported (26, 31).

To identify the unknown protein or complex in the purple band, protein identification was performed by nano-liquid chromatography–tandem mass spectrometry (nanoLC-MS/MS) after in-gel trypsin digestion of excised bands. The identified peptide sequences suggested that this band corresponds to a XOR family complex annotated putative mononuclear molybdenum carbon monoxide (CO) dehydrogenase of *R. gelatinosus*. Indeed, analysis of the MS data (Fig. 1B) showed the presence of the three subunits of a putative CO dehydrogenase (with a predicted mass of 126 kDa, encoded by the *coxSLM* operon) (Fig. 1C). CoxS is the small subunit encompassing two 2Fe-2S clusters (molecular mass, 17 kDa), CoxL corresponds to the large subunit (Moco subunit) (molecular mass, 84 kDa), and CoxM corresponds to the medium (FAD-containing) subunit (molecular mass, 27 kDa). Interestingly, CoxI (molecular mass, 35 kDa) was also detected and shares similarities with the XdhC protein required for the Moco cofactor insertion and the proper folding of the XOR enzyme (32).

### The *coxSLM* operon encodes the NBT-reducing complex in *R. gelatinosus*.

**FIG 2:**
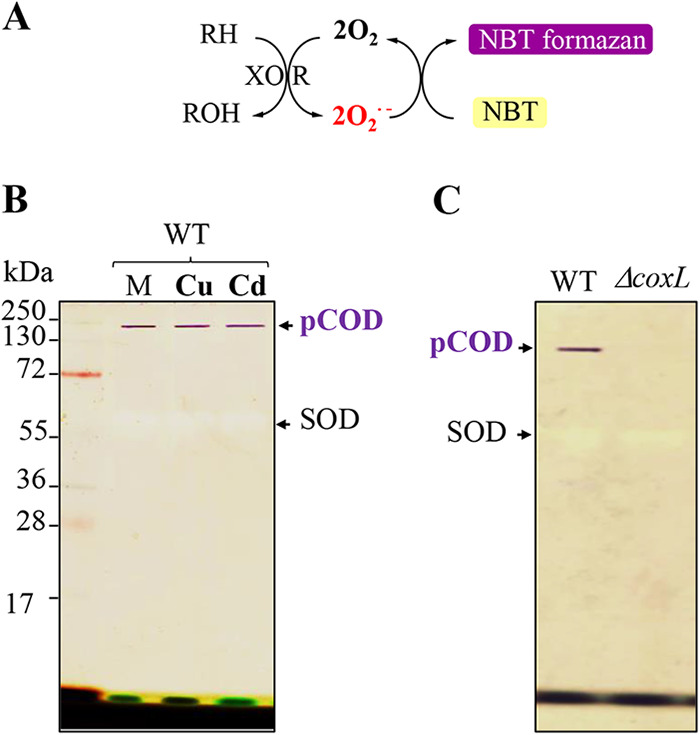
Identification of the putative CO dehydrogenase pCOD in *R. gelatinosus.* (A) Schematic representation of the reaction performed by the xanthine oxidoreductases leading to the purple precipitate observed upon NBT reduction in the absence of any external superoxide ion producer. (B) Soluble protein fractions of the WT strain grown in malate medium (M), malate medium supplemented with 100 μM CuSO_4_ (Cu), or malate medium supplemented with 100 μM CdCl_2_ (Cd). The same amount of proteins (40 μg) was separated on 12% non-denaturing PAGE gels. An in-gel activity assay was performed as described in Materials and Methods to reveal the pCOD. (C) Soluble fractions of WT and Δ*coxL* strains grown in malate medium were separated, and the pCOD activity assay was performed as described for panel B.

To unambiguously demonstrate that the NBT-reducing active band of *R. gelatinosus* corresponds to the complex encoded by the *coxSLM* operon as suggested by the MS analysis, we disrupted the operon by the insertion of a trimethoprim (Tp) resistance cassette within the coding region of *coxL*. Subsequently, we assayed for the presence of the dark purple active band within the soluble protein extracts. During the previously described in-gel SOD activity assay, riboflavin was used as a source of free radicals (Fig. 2A), which does not allow us to formally confirm that the putative carbon monoxide dehydrogenase complex itself produces superoxide and reduces NBT. We therefore omitted riboflavin from subsequent in-gel assays. As shown in Fig. 2B, whether wild-type cells were untreated or exposed to excess CuSO_4_ or CdCl_2_, the putative complex was present in the soluble fractions and able to reduce NBT to NBT-formazan in the absence of riboflavin. Under these experimental conditions, the PAGE gel remained unstained and only a faint achromatic band corresponding to SOD could be observed (Fig. 2B). These data demonstrate that *R. gelatinosus* expressed a high-molecular-weight complex able to reduce NBT. It was also concluded that its activity was not abolished by 100 μM Cu^+^ or Cd^2+^ in the wild-type background.

When the soluble fractions of the wild-type and Δ*coxL* strains were compared on the same PAGE gel, the NBT-reducing band was present in the wild-type and absent in the Δ*coxL* sample (Fig. 2C), thereby confirming that the NBT-reducing complex is encoded by the *coxSLM* operon. The CoxSLM complex will be here referred to as pCOD for “putative CO dehydrogenase,” since the function of the complex to oxidize CO or reduce CO_2_ has not yet been demonstrated.

### *In vivo* and *in vitro* inhibition of the pCOD complex activity by excess Cu in *R. gelatinosus*.

**FIG 3:**
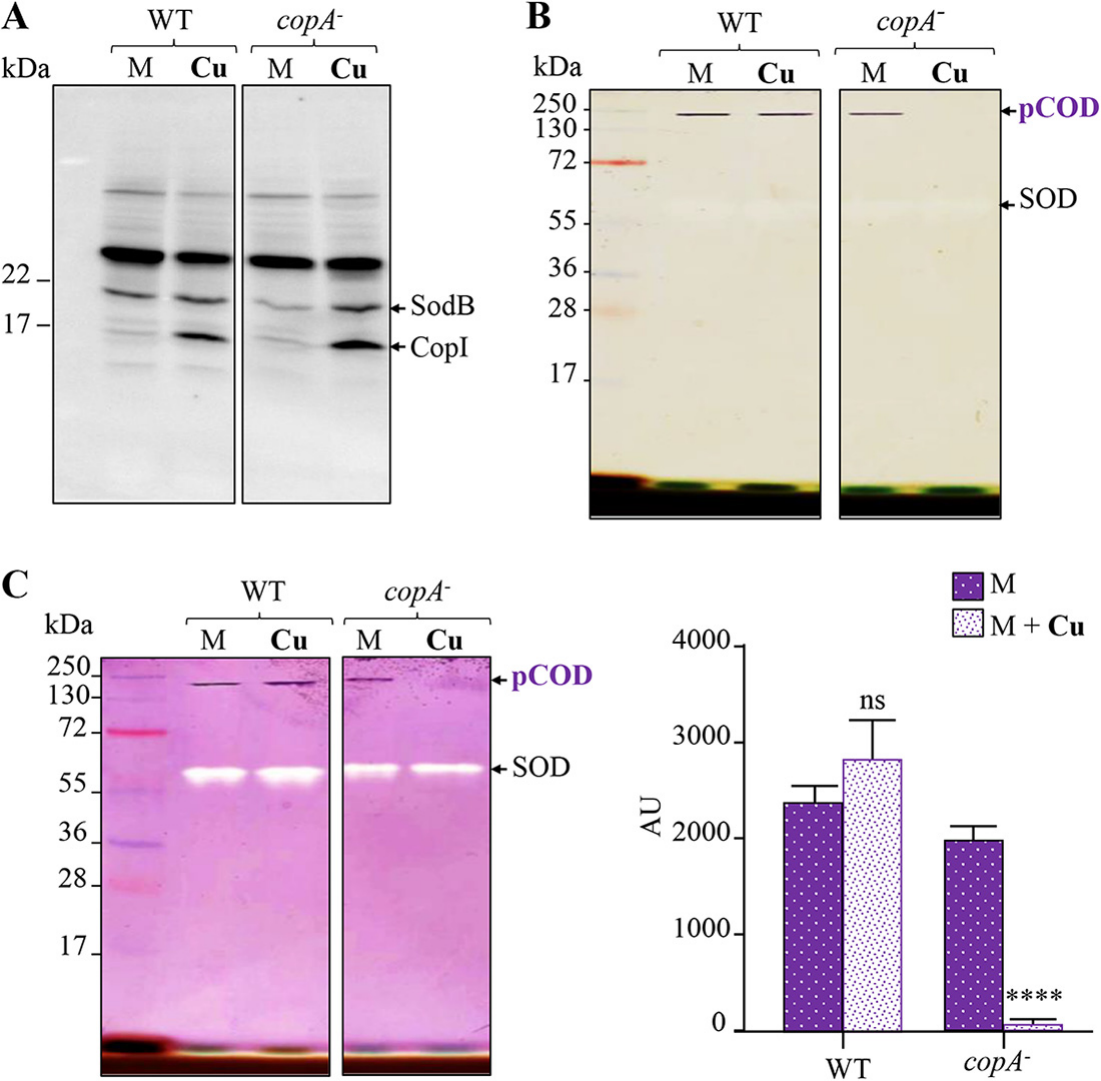
pCOD sensitivity to copper *in vivo*. (A) Western blot analysis (copy from Fig. 1A) of soluble protein fractions of the WT and the Cu^+^-efflux *copA*-minus mutant grown in malate medium (M) or malate medium supplemented with 100 μM CuSO_4_ (Cu). Induction of CopI is a marker of copper excess stress and response. (B) pCOD activity in the soluble protein fractions of the WT (copy from Fig. 2B) and the *copA*-minus mutant. (C) pCOD and SOD activities in the same soluble protein fractions from the WT (copy from Fig. 1A) and the *copA*-minus mutant and quantification of the in-gel pCOD activities. AU, arbitrary units. Data are presented as the mean ± standard deviation (SD) from 3 independent experiments. Statistical significance for this and all subsequent analyses was calculated using an ordinary one-way ANOVA (ns, not significant; *, *P* < 0.05; **, *P* < 0.01; ***, *P* < 0.001; ****, *P* < 0.0001).

The in-gel NBT reductase activity of the pCOD was previously unaffected by the presence of CuSO_4_ (Fig. 1A). Since the Cu^+^ detoxification (CopA/CopI) system (21, 31) in the WT had been induced (as shown by increased abundance of CopI on a Western blot [Fig. 1B]), the effect of Cu excess on pCOD activity in the WT background could be underestimated. Because the induced efflux ATPase CopA expels Cu^+^ outside the cytoplasm, it renders the analysis of excess Cu^+^ effects irrelevant in this background. We therefore took advantage of a mutant devoid of the Cu^+^-efflux ATPase CopA that accumulates Cu^+^ in the cytoplasm (21) to measure the effect of cytoplasmic excess Cu^+^ on pCOD activity. For that purpose, the WT and *copA*-minus mutant cells, which withstand the sublethal concentration of 100 μM CuSO_4_ (21), were grown in medium supplemented with or without 100 μM CuSO_4_, and the resulting soluble fractions of these cells were analyzed as done previously. To confirm the Cu^+^ stress, Fig. 3A shows the induction of CopI in response to Cu^+^ excess in WT and *copA*-minus strains. The in-gel activity assay confirmed that for the WT strain, Cu^+^ does not affect the activity of pCOD. The controls (WT) from Fig. 3 are from the same experiments as those shown in Fig. 1 and 2 and are thus being reused. In contrast, for the *copA*-minus mutant, addition of CuSO_4_ to the medium resulted in complete loss of the NBT reductase activity, in contrast to the SOD (Fig. 3B and C). These results showed that excess cytoplasmic Cu^+^ can inhibit *in vivo* the activity of the pCOD in *R. gelatinosus*.

**FIG 4:**
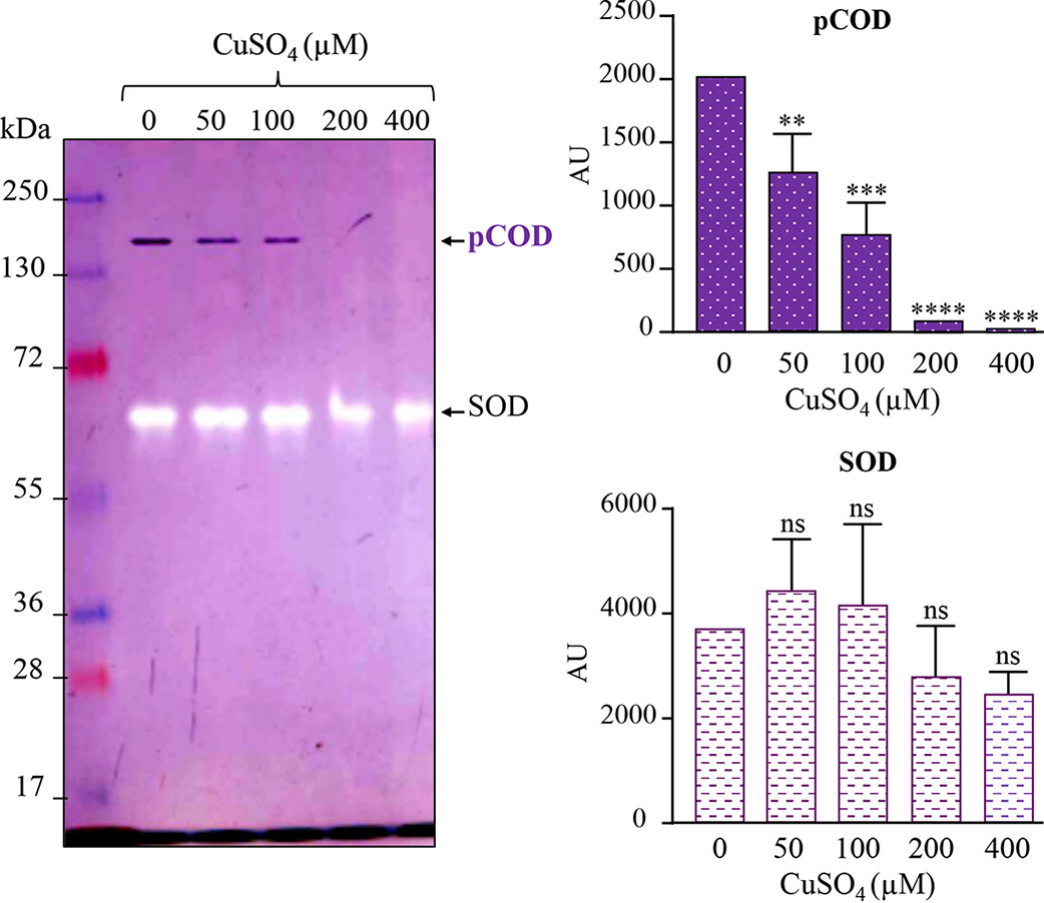
pCOD sensitivity to copper *in vitro*. Effect of CuSO_4_ treatment on pCOD and SOD activities in the soluble fraction of WT cells grown in malate medium. 40 mg/ml of soluble proteins was mixed with increasing concentrations of CuSO_4_ (0 to 400 μM) for 30 min at room temperature. Cu-treated proteins were then separated on 10% non-denaturing PAGE gels to assay for the pCOD and SOD activities. For quantification of the in-gel activities, data are presented as the mean ± SD from 3 independent experiments.

The results also raised the question whether Cu^+^ inhibits the assembly or the activity of the enzyme. Indeed, direct Cu^+^ inhibition of Moco synthesis (9, 25) or Fe-S cluster biogenesis (33, 34) has been reported previously. This inhibition of synthesis could be at the origin of the observed copper effect on pCOD. To answer this question, we analyzed *in vitro* the effect of CuSO_4_ treatment on the NBT reductase activity of the pCOD complex in the soluble protein fraction. For that purpose, soluble proteins from WT cells were incubated in buffer supplemented with increasing concentrations of CuSO_4_ (from 1.6 to 2,000 μM) for 30 min at room temperature. In this experiment, we used the SOD activity assay as a control to simultaneously compare the effects of excess CuSO_4_ on pCOD and on SOD. Similar to the *in vivo* data, incubation of soluble proteins with increasing concentration of CuSO_4_ led to a decreased activity of the pCOD complex in a concentration-dependent manner (Fig. 4). In contrast, excess Cu^+^ only marginally affected the activity of the SOD during the *in vitro* assay. Altogether, these data showed that Cu^+^ directly inhibits the activity of the assembled pCOD complex.

### Cadmium also inhibits the pCOD complex activity.

**FIG 5:**
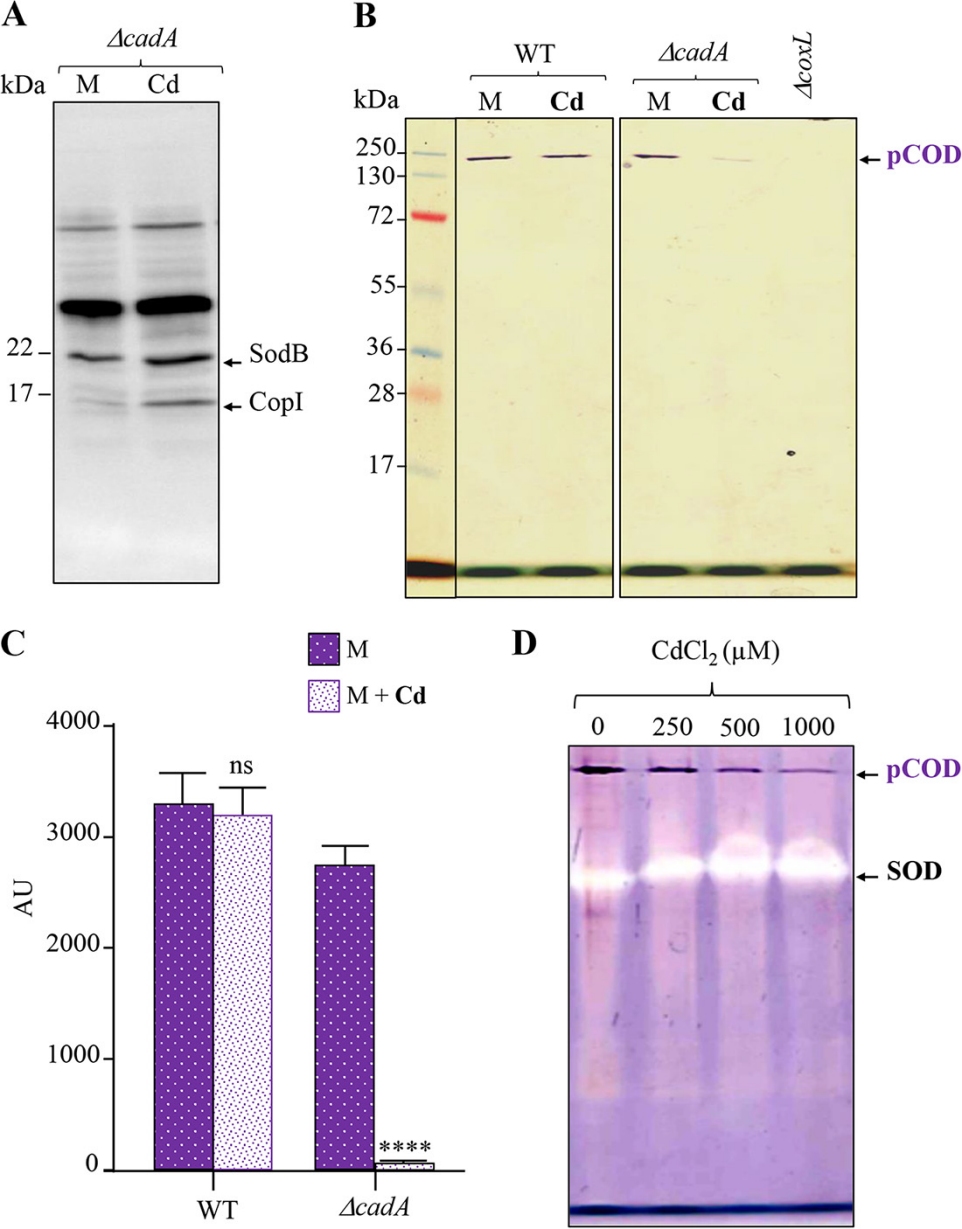
pCOD sensitivity to cadmium *in vivo* and *in vitro*. (A) Western blot analysis of soluble protein fractions of the WT and the Cd^2+^-efflux Δ*cadA* mutant grown in malate medium (M) or malate medium supplemented with 100 μM CdCl_2_ (Cd). Induction of CopI is also a marker of cadmium stress and response. (B) pCOD activity in the soluble protein fractions of the WT and Δ*cadA* mutant. pCOD activity was inhibited only in the Δ*cadA* mutant under Cd stress. (C) pCOD activity quantification from the gels in panel B. Data are presented as the mean ± SD from 3 independent experiments. (D) Effect of CdCl_2_ treatment on pCOD and SOD activities in the soluble fraction of WT cells grown in malate medium. Forty milligrams per milliliter of soluble proteins was mixed with increasing concentrations of CdCl_2_ (0 to 1,000 μM) for 30 min at room temperature. The same amount of proteins was then separated on a 12% non-denaturing PAGE gel to assay for the pCOD and SOD activities.

As copper is biologically and redox active, we questioned whether a redox-nonactive toxic metal such as cadmium could inhibit the activity of the pCOD complex of *R. gelatinosus*. To address this question, we used the mutant devoid of the Cd^2+^-efflux ATPase CadA to assess the effect of cytoplasmic accumulation of Cd^2+^ on the pCOD activity. While WT *R. gelatinosus* can tolerate up to 3 mM CdCl_2_ in its growth medium, the Δ*cadA* mutant is limited to low concentrations of up to 200 μM (26). In subsequent assays, the WT and Δ*cadA* mutant cells were grown in the presence of 100 μM CdCl_2_. The activity of pCOD in the soluble fractions of these cells was then analyzed by non-denaturing PAGE as described above. The in-gel activity confirmed that for the WT strain, Cd^2+^ does not affect the activity of pCOD, very likely because Cd^2+^ is expelled outside the cytoplasm. To confirm the Cd^2+^ stress, Fig. 5A shows the induction of CopI in response to Cd^2+^ excess in a Δ*cadA* strain as previously reported (26). In contrast, for the Δ*cadA* mutant, addition of 100 μM CdCl_2_ to the medium resulted in a drastic loss of the NBT reductase activity (Fig. 5B and C). These results clearly showed that Cd^2+^ can inhibit the activity of the *R. gelatinosus* pCOD complex *in vivo*. Similarly, to show that Cd^2+^ affects the assembled pCOD complex, we analyzed the *in vitro* effect of CdCl_2_ on the NBT reductase activity of the pCOD complex and on the SOD activity in enriched soluble protein fractions. In this experiment, soluble proteins from WT cells were incubated in buffers supplemented with increasing concentrations of CdCl_2_ (from 0 to 2,000 μM) at room temperature for 30 min. Similar to the *in vivo* data, incubation of soluble proteins with increasing concentrations of CdCl_2_ led to a decline in the activity of the pCOD complex (Fig. 5D), while cadmium did not affect the activity of the SOD, similarly to copper excess. We should note, however, that the effect of Cd^2+^ *in vitro* was not as strong as the effect of Cu^+^ (Fig. 4). Nevertheless, taken together the data strongly suggested that Cd^2+^ directly inhibits the activity of the assembled pCOD complex.

### Quantitative differential proteomics confirms pCOD complex susceptibility to Cu and Cd.

In an independent study, we sought to identify metal stress-responsive and/or target proteins in *R. gelatinosus*, through a quantitative differential shotgun proteomics (35) analysis of cells grown either with 100 μM CuSO_4_ or 100 μM CdCl_2_ or under FeSO_4_-limited conditions (unpublished data). Here, we extracted the specific peptide signals of the different subunits of the pCOD complex and compared the abundances of these proteins in WT, *copA*-minus, and Δ*cadA* cells grown in the presence or absence of metals. The abundance of the three subunits decreased significantly by as much as 17-fold for CoxL, 4-fold for CoxS, and 13-fold for CoxM in the Cu^+^-efflux ATPase mutant *copA*-minus strain, when exposed to 100 μM CuSO_4_, compared to their abundance in unexposed control cells (Table 1). Similar fold decreases in the abundance of CoxLMS proteins were also observed in the Δ*cadA* mutant in the presence of 100 μM CdCl_2_, compared to the unexposed control cells. Conversely, but as expected, proteins required for metal homeostasis, such as CopA, CopI, CopR, and CadA, were strongly increased in abundance in stressed cells compared to the control (Table 1). These data confirmed that excess Cu^+^ and Cd^2+^ affected the amount of pCOD complex subunit, in agreement with the decrease or loss of its activity in cells facing excess Cu^+^ or Cd^2+^.

**TABLE 1:** Selected proteins whose abundances were affected under Cu or Cd excess growth conditions^*a*^

Protein accession	Functional description	Tfold^*b*^	
		+Cu^*c*^	+Cd^*d*^
Decrease of the putative CO dehydrogenase			
RGS1_v6_1519|ID:50970044|CutL/CoxL|	Carbon monoxide dehydrogenase large chain (Rubrivivax gelatinosus)	−17.50	−20.42
RGS1_v6_1518|ID:50970043|CoxS|	Carbon monoxide dehydrogenase small chain (Rubrivivax gelatinosus)	−4.33	−2.17
RGS1_v6_1520|ID:50970045|CoxM|	Carbon monoxide dehydrogenase medium chain (Rubrivivax gelatinosus)	−13.80	−3.83
Induced proteins involved in metal tolerance			
RGS1_v6_1224|ID:50969749|CopA|	Copper ATPase (Rubrivivax gelatinosus)	44.00	50.00
RGS1_v6_1227|ID:50969752|CopI|	CopI (Rubrivivax gelatinosus)	5.54	5.31
RGS1_v6_1228|ID:50969753|CopJ|	CopJ (Rubrivivax gelatinosus)	27.33	1.67
RGS1_v6_1225|ID:50969750|CopR|	HTH^*e*^-type transcriptional regulator HmrR (Rubrivivax gelatinosus)	12.67	11.67
RGS1_v6_3025|ID:50971550|CadA|	Heavy-metal-translocating P-type ATPase (Rubrivivax gelatinosus)	2.75	6.25

a Cox (putative carbon monoxide dehydrogenase) subunits decreased, while proteins involved in efflux and metal tolerance were increased.

b The Tfold was calculated by dividing the sum of the spectral counts to which the value 3 was added in the first condition (because 3 replicates) by the sum of the spectral counts in the other condition to which the value 3 was added. A minus sign is shown when the conditions must be inverted to obtain a ratio greater than 1.

c Tfold for *copA*-minus malate medium with copper versus WT malate medium.

d Tfold for Δ*cadA* malate medium with cadmium versus WT malate medium.

e HTH, helix-turn-helix.

### Identification of XDH of *R. gelatinosus*.

**FIG 6:**
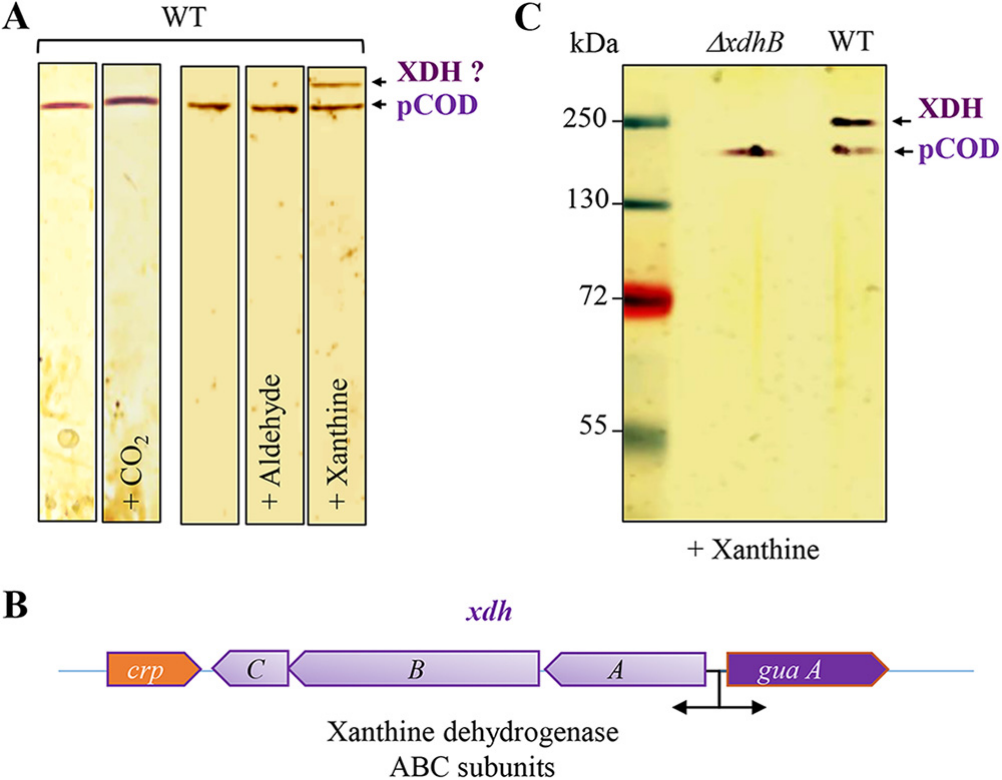
Identification of xanthine dehydrogenase (XDH) in *R. gelatinosus.* (A) Soluble protein fractions of the WT strain grown in malate medium were separated on 10% non-denaturing PAGE gels. The XOR in-gel activity assay was performed as described in Materials and Methods with the addition of aldehyde or xanthine or bubbling with CO_2_. The assay revealed the XDH active band after addition of xanthine. (B) Graphic description of the *xdhABC* operon encoding the three subunits of the xanthine dehydrogenase of *R. gelatinosus*. *guaA* corresponds to a gene encoding a GMP synthase (glutamine hydrolyzing), and *crp* encodes a putative catabolite repressor protein. (C) Soluble fractions of WT and Δ*xdhB* strains grown in malate medium were separated, and the in-gel assay for activity was performed in the presence of xanthine to reveal pCOD and XDH.

Non-denaturing PAGE preserves the native state of protein complexes, enabling subsequent identification using activity assays. To identify the substrate of the newly identified pCOD complex, we tested different molecules for their ability to significantly increase the activity of the pCOD-containing polyacrylamide band (Fig. 6A). Different aldehydes (formaldehyde, acetaldehyde, *n*-octyl-aldehyde, and butyraldehyde) did not change the activity of the pCOD complex. In CO_2_-enriched growth medium, the activity of the pCOD was slightly increased, suggesting that the pCOD could have a CO/CO_2_ dehydrogenase/reductase activity. Yet, further experiments are required to confirm this observation. Interestingly, the addition of xanthine to the native PAGE gel revealed a second active band of approximately 250 kDa. These data strongly suggested that this new complex corresponds to the xanthine dehydrogenase (XDH) of *R. gelatinosus*. An operon encompassing three genes (*xdhABC*) that could encode the three subunits of the xanthine dehydrogenase in *R. gelatinosus* was therefore cloned from the genome (Fig. 6B). To identify the activity of the complex encoded by these genes, an Δ*xdhB* deletion mutant was generated. Soluble fractions from the WT and the Δ*xdhB* mutant were assayed for the presence of the active band in the presence of xanthine as the substrate. As shown in Fig. 6C, the pCOD band was revealed in both samples, while the band corresponding to the XDH was absent in the Δ*xdhB* deletion mutant, suggesting that the 250 kDa complex corresponds to the XDH complex. According to the theoretical molecular weight of the three subunits (XdhB, 82 kDa; XdhA, 54 kDa; and XdhC, 27 kDa), the XDH complex would be a homodimer on the native gel, which has been observed for other bacterial and mammalian XDH enzymes.

### Excess Cu or Cd also inhibits bacterial and mammalian XDH complex activity.

**FIG 7:**
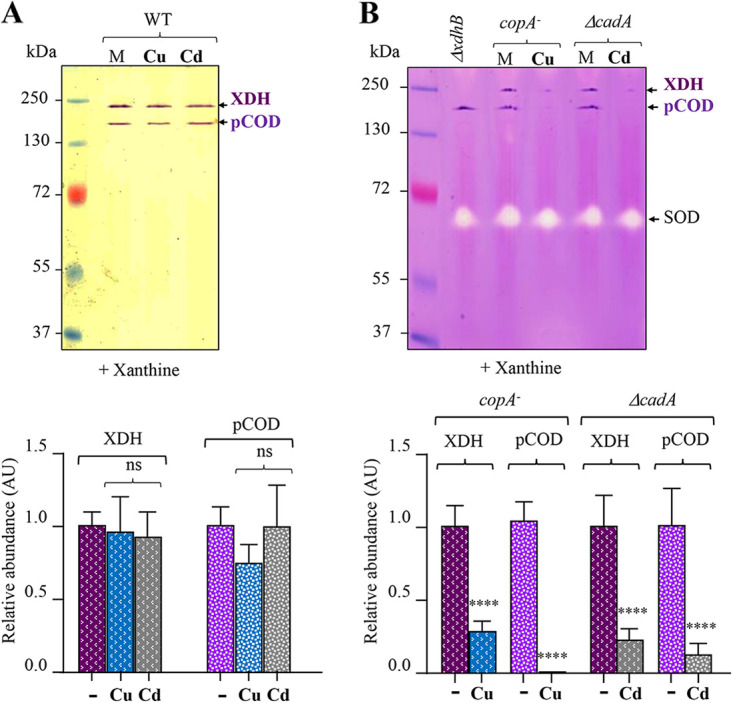
XDH sensitivity to copper and cadmium *in vivo*. (A) pCOD and XDH activities in the soluble protein fraction of the WT cells grown in malate medium (M) or malate medium supplemented with 100 μM CuSO_4_ (Cu) or 100 μM CdCl_2_ (Cd). (B) pCOD, XDH, and SOD activities in the soluble protein fractions of the *copA*-minus and Δ*cadA* mutants grown in malate medium (M) or malate medium supplemented with 100 μM CuSO_4_ (Cu) or 100 μM CdCl_2_ (Cd). Only pCOD and XDH activities were inhibited in the mutants under Cu or Cd stress. For quantification of the in-gel activities (lower panels), data are presented as the mean ± SD from 3 independent experiments.

**FIG 8:**
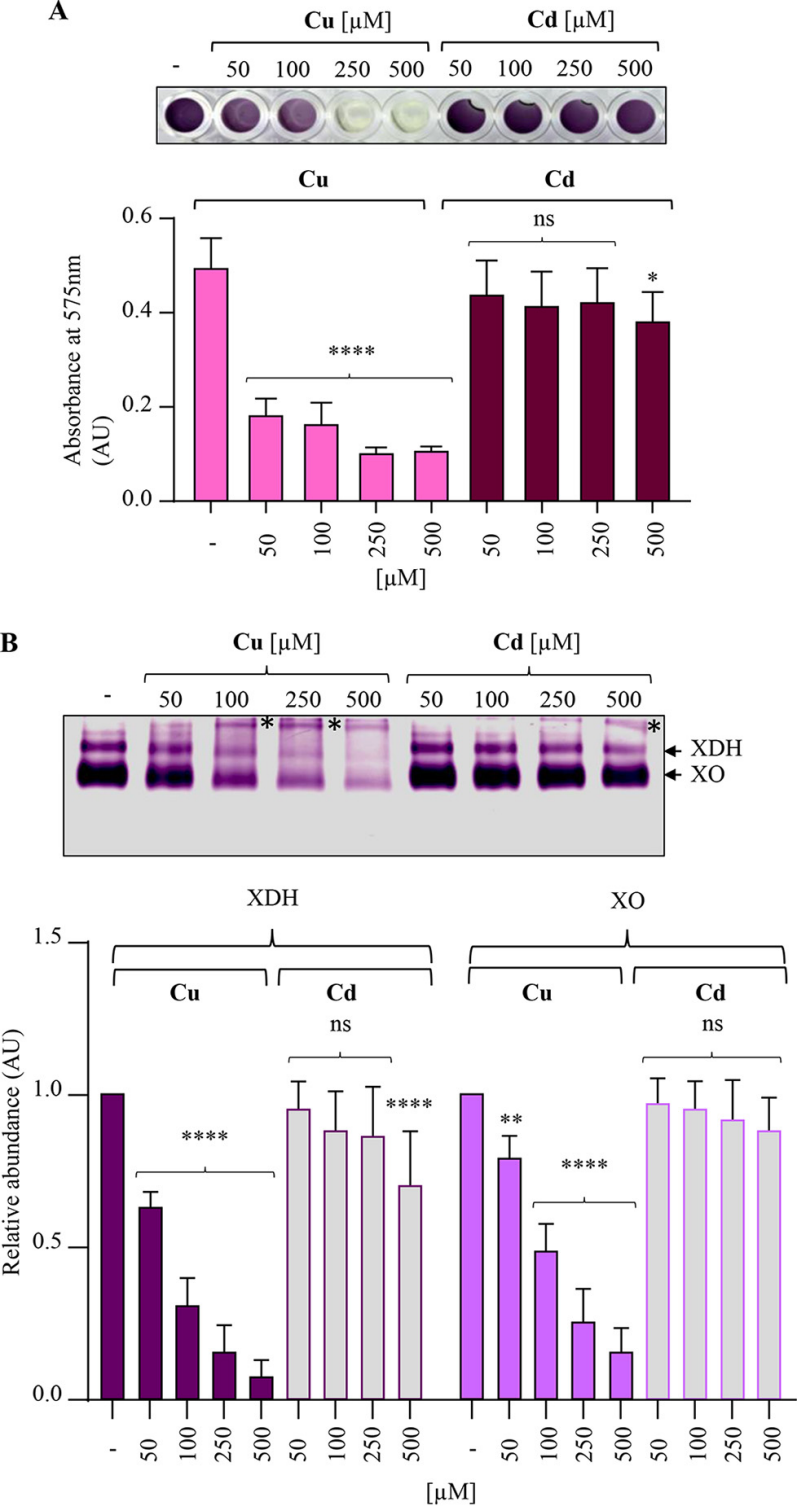
Mammalian XOR (XDH/XO) sensitivity to copper and cadmium. (A) Microtiter plate-based xanthine oxidase assay. One hundred microliters of bovine milk XOR (0.01 U/mL) was incubated with or without (−) increasing CuSO_4_ (Cu) or CdCl_2_ (Cd) for 30 min prior to addition of xanthine. (B) Bovine milk XOR was incubated in the buffer without (−) or with increasing CuSO_4_ (Cu) or CdCl_2_ (Cd) for 30 min. Cu- or Cd-treated XOR was then separated on 10% non-denaturing PAGE gels to assay for the XOR activity. Asterisks on the gel indicate protein aggregates. For quantification of the in-gel activities, data are presented as the mean ± SD from 3 independent experiments.

It has been reported that compounds containing copper or zinc are potential inhibitors of xanthine XOR/XDH activity *in vitro* using mouse liver tissue homogenate (36). However, direct evidence showing an inhibitory effect of metals on XDH activity *in vivo* was still lacking. We therefore tested the effect of excess Cu^+^ or Cd^2+^ on the activity of the cytosolic XDH *in vivo* by growing WT, *copA*-minus, or Δ*cadA* mutant *R. gelatinosus* cells with or without excess Cu or Cd. The soluble fractions were analyzed on non-denaturing PAGE gels for XDH NBT reductase activity. In the WT background, XDH activity was unaffected by the addition of Cu or Cd (Fig. 7A), similarly to pCOD activity. In contrast, in the strains that accumulate Cu^+^ or Cd^2+^, the activities of both pCOD and XDH were strongly inhibited by both ions (Fig. 7B). These data clearly showed that Cu^+^ and Cd^2+^ inhibit the activity of different enzymes in the bacterial XDH/XOR family. We also sought to verify whether mammalian XDH activity could be compromised by exposure to Cu^+^ or Cd^2+^. In mammalian cells, xanthine oxidase (XO) and xanthine dehydrogenase (XDH) are interconvertible forms of the same enzyme, known as xanthine oxidoreductase (XOR) (28, 37). The xanthine oxidoreductase activity of bovine milk XOR was tested either in liquid or by non-denaturing PAGE in the presence of xanthine with increasing concentrations of CuSO_4_ or CdCl_2_. During the in-liquid reactions, as expected the untreated samples showed the formation of a dark blue color formed due to the interaction of superoxide ions with NBT (Fig. 8A). Low concentrations of CuSO_4_ in the reaction buffer generated a light blue color, demonstrating a decrease in the enzyme activity. No color was developed in the samples exposed to 250 or 500 μM CuSO_4_. Similarly, on the non-denaturing PAGE gel, CuSO_4_ affected the activity of the enzymes (XO and XDH) in a concentration-dependent manner (Fig. 8B). We also noticed the presence of protein aggregates (high-molecular-weight bands) in the presence of metals. In both assays, copper effectively inhibited the XOR activity, but the mammalian XOR was not affected or only very slightly affected by Cd^2+^ under these assay conditions, in contrast to the bacterial XDH. Altogether, our data thoroughly support the inhibition of pCOD and XDH by excess Cu or Cd, thus illustrating the harmful effect of metals on the XOR family enzymes.

## DISCUSSION

The high toxicity of Cu, Cd, Pb, As, and other metals in bacteria arises from their ability to cross membranes and target various metalloenzymes and vital processes such as heme or Fe-S biosynthesis (1, 22, 31, 38). The vulnerability of metalloenzymes very often stems from the accessibility of their cofactors to these ions, which could lead to cofactor displacement or mismetalation. Accordingly, many enzymes whose cofactors are solvent exposed are likely to be targets of metals (22). Therefore, we can expect that metals could poison multiple metabolic pathways and disrupt the activity of many other not-yet-identified metalloenzymes. In this regard, using a single-step in-gel activity assay, we showed *in vivo* and *in vitro* that two (pCOD and XDH) members of the xanthine oxidoreductase family are inhibited by Cu and Cd (3, 4). Given the conservation of the structure of the XOR family enzymes, we suggest that metals could poison other enzymes in this family such as purine hydroxylases or aldehyde oxidases.

*In vivo*, inhibition could be the consequence of a direct effect of metals on XOR activity or assembly or even on the synthesis of the XOR cofactors, i.e., Moco and/or Fe-S clusters. Indeed, in E. coli, Cu^+^ or Cd^2+^ ions can be inserted nonspecifically into the molybdopterin (MPT) instead of molybdenum (Mo^2+^) (9). This resulted in the inhibition of molybdoenzymes such as sulfite oxidase by high concentrations of Cu^+^ or Cd^2+^ (9). Similarly, in the nitrogenase assembly pathway, Cu^+^ almost completely abolished Mo transfer from NifQ to NifENH (39). An additional threat posed by Cu^+^ and Cd^2+^ *in vivo* was proposed to occur on the Fe-S cluster-containing proteins (21, 22, 33, 34). However, the two 2Fe-2S clusters of the XDH or the COD complexes may not be impacted by Cu^+^ or Cd^2+^ since the clusters are buried and inaccessible to solvent (10, 40). Until now, only exposed 4Fe-4S clusters were identified as targets of these metals within dehydratases (21, 22, 26). Nonetheless, Cu^+^ and Cd^2+^ appear to bind and inhibit components of the bacterial ISC or SUF Fe-S biogenesis machinery (33, 34), which could affect the assembly of XOR. A direct effect is also possible; indeed, the crystal structure of R. capsulatus XDH revealed a direct coordination of the substrate with the Mo ion of the Moco and revealed that water molecules are positioned close to the Moco cluster (10). It follows that access to the molybdenum center by ions in the solvent is possible and could account for the inhibition of XOR by Cu^+^ or other ions. Binding of arsenite (AS) to the molybdenum center of bovine xanthine oxidase also inhibits the enzyme activity (15, 41). The crystallographic structure of the As-inhibited XOR from Desulfovibrio gigas revealed a double bridge between Mo and As in the active site of the enzyme, thus providing a structural basis to account for the inhibition. Finally, it should be emphasized that Cu^2+^ induced XOR protein aggregates in our *in vitro* experiments (Fig. 8B). The ability of Cu to bind and induce protein aggregation *in vivo* was recently reported in E. coli (42) and other organisms (43). This should be also taken into account to explain in part the inhibitory effect of Cu on XORs.

*In vitro*, our data showed also that Cu or Cd inhibits the activity of assembled and active XOR complexes. A possible explanation is that Cu^+^ or Cd^2+^ can displace/replace the Mo^2+^ atom from Moco and interact directly with the MPT dithiolene sulfurs. The fact that the enzymes remained inhibited after the in-gel electrophoresis suggests either that the metal remains associated with the enzyme or that it displaces the Moco. Replacement of Mo^2+^ by Cu^+^ or Cd^2+^ within the Moco center has been demonstrated (9). In the plant Cnx1 protein that catalyzes the insertion of Mo^2+^ into the MPT moiety, Cu^+^ was bound to the MPT dithiolate sulfurs in the structure of Cnx1 (25) and it inhibited the Cnx1 activity. Similarly, expression of the human sulfite oxidase in E. coli, in the presence of 100 μM Cu^+^ or Cd^2+^, resulted in the insertion of these metals in the MPT of the protein (9). The *in vitro* study suggested that the nonspecifically produced Cu-MPT or Cd-MPT can be inserted into MPT-binding molybdoenzymes and can contribute to the toxicity of Cu^+^ or Cd^2+^ in E. coli (9).

Copper-based therapeutic agents have a long history of use in the treatment of disease. Copper has been used since the mid-19th century as a treatment for anemia (44). More recently, in Menkes disease and ATP7A-related symptoms, copper histidine or copper chloride injections were effective in improving developmental and neurological issues in patients (45, 46). Gout arthritis is caused by excessive concentrations of uric acid in the body. Drugs have been discovered which treat gout through the inhibition of xanthine oxidoreductase-mediated uric acid generation. However, new and efficient therapy is required to overcome the problems of resistance or side effects. Here, we show that the activity of the bacterial and bovine XORs could be reduced or abolished by copper ions. Targeting the mammalian xanthine oxidoreductase by copper could be a promising alternative to modulating XOR activity. In agreement with this, it was reported that Cu or Cu-containing compounds significantly inhibit XDH activity and reduce uric acid generation by the mouse liver homogenate XDH (13, 36).

## MATERIALS AND METHODS

### Bacterial strains and construction of mutants.

*R. gelatinosus* S1 (47) was grown overnight at 30°C in malate medium under photosynthetic condition (light and microaerobiosis, in filled tubes with residual oxygen in the medium). E. coli was grown overnight at 37°C in LB medium. Antibiotics were added at the indicated concentrations when appropriate: kanamycin (50 μg/mL) and trimethoprim (50 μg/mL). Bacterial strains and plasmids used in this study are listed in Table 2. Standard methods were performed according to the work of Sambrook et al. (48) unless indicated otherwise. To inactivate *coxL*, a 2.2-kb fragment was amplified using the primers coxLF (5′-CTACCAGAACATCGTCAAGG-3′) and coxLR (5′-CATCGTGTAGTCCATGTAGC-3′) and cloned into the PCR cloning vector pGEM-T to give pGcoxL. The *coxL* gene was then inactivated by deletion of a 279-bp BglII fragment and the insertion of the 0.7-kb Tp cassette (49) at the BglII site within the *coxL* coding sequence. The resulting recombinant plasmid was designated pGcoxL::Tp. This plasmid was used to transform the WT strain. To disrupt *xdhB*, a 1,711-bp fragment was amplified using the primers xdhBF (5′-CAACTGGCCGAGATGTGGCG-3′) and xdhBR (5′-CCAGTAGCAGTTGTCGAAGT-3′) and cloned into the PCR cloning vector pGEM-T to give pGxdhB. The *xdhB* gene was then inactivated by insertion of the 0.7-kb Tp cassette at the MscI site within the *xdhB* coding sequence. The resulting recombinant plasmid was designated pGxdhB::Tp. This plasmid was used to transform the WT strain. Transformants were selected on malate plates supplemented with the appropriate antibiotics (trimethoprim or kanamycin) under photosynthesis conditions. Following transformant selection, template genomic DNA was prepared from the ampicillin-sensitive transformants and confirmation of the antibiotic resistance marker’s presence at the desired *coxL* or *xdhB* locus was performed by PCR.

**TABLE 2:** Bacterial strains and plasmids

Strain or plasmid	Relevant characteristic(s)^*a*^	Source or reference
Strains		
E. coli JM109	*el4* mutant (McrA^−^) *recA1 endA1 gyrA69 thi-1 hsdR17*(r_K_^−^ m_K_^+^) *supE44 reA1* Δ(*lac-proAB*) [F′ *traD36 proAB lacIZ*ΔM15]	Stratagene
*R. gelatinosus*		
S1	Wild-type strain	47
*copA*-minus mutant	*copA*-inactivated strain Km^r^ (*copA*::Tn*5*, Km)	21
Δ*cadA* mutant	*cadA*-inactivated strain Tp^r^ (Δ*cadA*::Tp)	26
Δ*coxL* mutant	*coxL*-inactivated strain Tp^r^ (Δ*coxL*::Tp)	This work
Δ*xdhB* mutant	*xdhB*-inactivated strain Tp^r^ (*xdhB*::Tp)	This work
Plasmids		
pGEM-T	Cloning vector (Ap^r^)	Promega
pUC4K	Plasmid bearing the Km cartridge (Ap^r^ Km^r^)	Pharmacia
p34S-Tp	Plasmid bearing the Tp cartridge (Ap^r^ Tp^r^)	49
pGcoxL	pGEM-T plus 2,198-bp PCR fragment containing coxL	This work
pGcoxL::Tp	Tp cartridge cloned into BglII sites of coxL in pGcoxL	This work
pGxdhB	pGEM-T plus 1,711-bp PCR fragment containing *xdhAB*	This work
pGxdhB::Tp	Km cartridge cloned into StuI site in cadR in pGcadR	This work

a Abbreviations: Ap^r^, ampicillin resistant; Km^r^, kanamycin resistant; Tp^r^, trimethoprim resistant.

### Soluble protein preparation.

Cells were disrupted by sonication in 50 mM Tris-HCl buffer (pH 7.6) containing 1 mM phenylmethylsulfonyl fluoride. Unbroken cells and membranes were removed by ultracentrifugation (200,000 × *g*, 90 min, 4°C) to collect the soluble fraction. Protein concentration was estimated using the bicinchoninic acid assay (Sigma), with bovine serum albumin as the standard. For soluble protein metal treatment, required concentrations of metal solution were mixed with a total protein concentration of 20 mg/mL at room temperature (RT) for 30 min.

### SOD and NBT reductase in-gel activity assays on non-denaturing gel electrophoresis gels.

Forty micrograms of soluble proteins was separated on a 12% non-denaturing polyacrylamide gel and stained for SOD activity as described in the work of Weydert and Cullen (29), with minor modifications. Nitroblue tetrazolium (NBT) (2 mg/mL) was added first for a 15-min incubation in the dark at RT followed by the addition of TEMED (*N*,*N*,*N*′,*N*′-tetramethylethylenediamine) (0.85%) and riboflavin-5-phosphate (56 μM) for 15 min in the light. The gel was washed twice in double-distilled water (ddH_2_O) and then left in ddH_2_O at RT on a light box until SOD-positive staining appeared. For NBT reductase activity, the gel was incubated in 50 mM Tris-HCl buffer (pH 7.6) supplemented only with NBT (2 mg/mL) for 15 to 30 min in the dark at RT.

### Mass spectrometry analyses.

Peptide mixtures generated by standard in-gel digestion with trypsin (Gold; Promega) were SpeedVac treated for 10 min and then analyzed with a quadrupole time of flight (QTOF) Premier mass spectrometer coupled to an Acquity nano-ultraperformance liquid chromatograph equipped with a trapping and an analytical column as described in reference 31.

### Whole-cell shotgun proteomics.

Bacterial pellets were diluted in 1× LDS (Thermo) buffer at a ratio of 7.5 μL/mg. They were heated for 10 min at 99°C, sonicated for 10 min, and then subjected to bead-beating lysis as previously described (50). The extracted proteins (25 μL) were subjected to denaturing electrophoresis onto a NuPAGE 4 to 12% Bis-Tris gel (Thermo) for 4 min. For each sample, a single gel band containing the whole cellular proteome was excised and processed as described previously (51) for in-gel trypsin proteolysis. Peptides were analyzed using a Q-Exactive HF tandem mass spectrometer (Thermo) coupled to an Ultimate 3000 nanoRSLC nanoLC system (Thermo) in data-dependent acquisition mode as previously described in reference 52.

### Microtiter plate-based milk bovine XOR assay.

XO from bovine milk was purchased from Sigma-Aldrich Co. (reference no. X1875; ≥0.4 units/mg protein units/mL) and diluted in 50 mM Tris-HCl buffer (pH 7.6). For the assay, 100 μL of XOR (0.01 U/mL) in the same buffer was mixed or not with increasing CuSO_4_ concentrations. After 30 min of incubation at room temperature, 50 μL of buffer containing NBT (2 mg/mL) and xanthine (2 mmol/L) was added to the XOR samples to start the XOR reaction.

### Western blotting and His-Probe-HRP detection.

Equal amounts of soluble proteins (20 μg) were separated on SDS-PAGE gels and transferred onto a Hybond ECL polyvinylidene difluoride membrane (GE Healthcare). The membrane was then probed with His-Probe-HRP (horseradish peroxidase; from Pierce) according to the manufacturer’s instructions. His-Probe-HRP allows detection of CopI and SodB, two naturally histidine-rich proteins in the *R. gelatinosus* soluble fraction (30). Positive bands were detected using a chemiluminescent HRP substrate according to the method of Haan and Behrmann (53). Image capture was performed with a ChemiDoc camera system (Bio-Rad).

### Quantification and statistical analyses.

The bands in the in-gel assay were quantified using the ImageJ program. The relative amount was calculated based on the signal obtained in the untreated sample. Statistical significance was calculated using an ordinary one-way analysis of variance (ANOVA)/GraphPad Prism (ns, not significant; *, *P* < 0.05; **, *P* < 0.01; ***, *P* < 0.001; ****, *P* < 0.0001).

### Data availability.

The mass spectrometry proteomics data have been deposited in the Figshare repository with the data set identifier https://doi.org/10.6084/m9.figshare.23615265.
